# Glutamate load fosters spreading depolarization under osmotic stress in brain slices

**DOI:** 10.3389/fncel.2025.1722194

**Published:** 2026-01-21

**Authors:** Rita Frank, Stephane Marinesco, Ferenc Bari, Ákos Menyhárt, Eszter Farkas

**Affiliations:** 1 Hungarian Centre of Excellence for Molecular Medicine, University of Szeged Cerebral Blood Flow and Metabolism Research Group, Szeged, Hungary; 2 Department of Cell Biology and Molecular Medicine, Albert Szent-Györgyi Medical School and Faculty of Science and Informatics, University of Szeged, Szeged, Hungary; 3 Lyon Neuroscience Research Center, Inserm U1028, CNRS UMR, University Claude Bernard Lyon I, Lyon, France; 4 Department of Medical Physics and Informatics, Albert Szent-Györgyi Medical School and Faculty of Science and Informatics, University of Szeged, Szeged, Hungary

**Keywords:** cerebral edema, spreading depolarization, astrocyte swelling, extracellular glutamate accumulation, brain slice

## Abstract

**Introduction:**

Cerebral edema is a hallmark of lesion progression after acute ischemic stroke (AIS) and a major contributor to the evolution of spreading depolarizations (SDs). SDs trigger extracellular glutamate accumulation and excitotoxic injury, yet the mechanisms linking edema formation, glutamate dysregulation, and SD dynamics remain incompletely understood. Here, we investigated how inhibiting glial swelling and volume-regulated glutamate release, or blocking neuronal ionotropic glutamate receptors alters SD features under hypo-osmotic stress in vitro.

**Methods:**

Acute 350-µm-thick brain slices were prepared from male Wistar rats (n = 24). Edema was induced using hypoosmotic medium (130→60 mM NaCl), and SD was triggered by hypoxia. SD evolution and extracellular glutamate levels were monitored using local field potential recordings, intrinsic optical signal imaging, and enzyme-based glutamate biosensors. Astrocyte swelling was reduced by blocking AQP4+NKCC1 (TGN-020 + bumetanide) and VRAC channels (DCPIB), while neuronal NMDA and AMPA/kainate receptors were antagonized with MK-801 + CNQX.

**Results:**

Inhibition of AQP4, NKCC1, or VRAC channels restricted the cortical area invaded by SD, shortened SD duration, and reduced extracellular glutamate accumulation. In contrast, blockade of NMDA or AMPA/kainate receptors markedly decreased SD propagation and glutamate buildup. Both astrocytic and neuronal interventions disrupted typical SD initiation patterns, producing atypical, multifocal SD events.

**Discussion:**

These findings demonstrate that astrocyte volume regulation and neuronal ionotropic glutamate receptors jointly shape SD characteristics under osmotic stress, identifying astrocytic water/ion homeostasis and glutamatergic signaling as potential therapeutic targets to limit excitotoxic injury in acute cerebrovascular disease.

## 1 Introduction

Cerebral edema is a major contributor to lesion expansion in cases of acute brain injury, including ischemic or hemorrhagic stroke, subarachnoid hemorrhage and traumatic brain injury(Simard et al., 2007; Stokum et al., 2016; Unterberg et al., 2004; Dreier et al., 2018). In the acute phase of these diseases, cytotoxic edema develops within minutes, characterized by cellular swelling that primarily affects neurons and astrocytes. This early, cytotoxic edema subsequently evolves into vasogenic edema, which is associated with blood brain barrier disruption and extracellular fluid accumulation(Risher et al., 2009; Unterberg et al., 2004). When malignant edema occurs—most notably with middle cerebral artery (MCA) infarction—progressive space-occupying swelling carries mortality rates up to 80% with conservative anti-edema treatment (Yang et al., 2015; Gul et al., 2018). Over the last decade, robust experimental studies proved that cerebral edema drives the evolution of spreading depolarizations (SDs), that are known to be key mediators of lesion progression (Dreier et al., 2018; Oka et al., 2022; Menyhart et al., 2022). SD is a wave of neuronal and glial depolarization that propagates through the cerebral grey matter causing transmembrane ion imbalance, glutamate release, followed by transient depression of neuronal activity and cytotoxic edema (Somjen, 2001; Dreier, 2011; Dreier et al., 2018). In concert with the earlier findings, we have demonstrated in brain slices, that osmotic stress accelerates the (i) speed of SD propagation and (ii) tissue swelling increases the focal area of SDs, thus leading to infarct growth (Menyhart et al., 2022). Given the evident association between the evolution of SDs and the development of cerebral edema, therapies should aim at modulating the cellular mechanisms underlying edema formation; however, the pathophysiological landscape remains highly complex and multifactorial.

Acute brain swelling often leads to glutamate release and uptake dysfunctions. ATP depletion makes neurons unable to maintain ion gradients across the cell membrane, leading to glutamate release from releasable stores, a process that is further aggravated by SDs. This results in glutamate accumulation and the development of glutamate excitotoxicity that triggers neuronal damage and lesion maturation. Although the concept of glutamate excitotoxicity was criticized for its questionable role in neuronal injury, the occurrence of SDs in the compromised, swollen tissue may be one of the mechanisms understood to cause glutamate accumulation to toxic levels to induce neuronal damage (Belov Kirdajova et al., 2020; Andrew et al., 2022).

The cellular contribution to glutamate excitotoxicity in brain edema is extremely complex. The primary effectors of excitotoxic glutamate accumulation are the ionotropic glutamate receptors, specifically the N-methyl-D-aspartate (NMDA) receptors and the α-amino-3-hydroxy-5-methyl-4-isoxazolepropionic acid (AMPA) receptors (Lau and Tymianski, 2010; Choi, 1988). Besides neurons astrocytes, have transporters that account for nearly 95% of the total glutamate uptake in the brain (Danbolt, 2001; Murphy-Royal et al., 2015). Furthermore, glial cells can become significant sources of glutamate when exposed to swelling, potentially triggering the evolution of SDs in the swollen tissue (Menyhart et al., 2022).

Therefore, we hypothesize that under hypo-osmotic stress, which resembles the early, cytotoxic edema observed after stroke, astrocyte swelling may represent a major driving force in SD evolution and extracellular glutamate accumulation, while neuronal ionotropic signaling causes glutamate accumulation irrespective of edema formation.

To test this hypothesis, we set out to investigate the mechanisms of SD-coupled glutamate buildup under osmotic stress by examining the roles of (i) glial swelling and volume-regulated glutamate release, and (ii) neuronal ionotropic glutamate receptor signaling.

## 2 Materials and methods

The experimental procedures were approved by the National Food Chain Safety and Animal Health Directorate of Csongrád-Csanád County, Hungary. The procedures were performed according to the guidelines of the Scientific Committee of Animal Experimentation of the Hungarian Academy of Sciences (updated Law and Regulations on Animal Protection: 40/2013. (II. 14.) Gov. of Hungary), following the EU Directive 2010/ 63/EU on the protection of animals used for scientific purposes and reported in compliance with the ARRIVE guidelines.

### 2.1 Acute brain slice preparations

Coronal brain slices were prepared as reported previously (Frank et al., 2021). Briefly, adult male Wistar rats (body weight: 250 g; *n* = 24) were deeply anesthetized with 5% isoflurane (in N_2_O: O_2_; 2:1), the animals were decapitated, 350 μm thick coronal brain slices anterior to bregma were cut with a vibrating blade microtome (Leica VT1000S, Leica, Germany) and collected in ice cold, modified artificial cerebrospinal fluid (aCSF; 130 NaCl, 3.5 KCl, 1 NaH_2_PO_4_, 24 NaHCO_3_, 1 CaCl_2_, 3 MgSO_4_ and 10 D-glucose in mM concentrations). Three to five slices were allowed to recover in carbogenated (95% O_2_, 5% CO_2_) normal aCSF (130 NaCl, 3.5 KCl, 1 NaH_2_PO_4_, 24 NaHCO_3_, 3 CaCl_2_, 1.5 MgSO_4_ and 10 D-glucose in mM concentrations; osmolarity: 339 mosm/l). Randomly selected slices were placed into an interface type recording tissue chamber (Brain Slice Chamber BSC1, Scientifc Systems Design Inc., Ontario, Canada), and were continuously perfused with carbogenated aCSF at a rate of 2.5 mL/min. Chamber temperature was maintained at 32 °C using a dedicated proportional temperature controller unit (PTC03, Scientifc Systems Design Inc., Ontario, Canada).

### 2.2 Hypo-osmotic cerebral edema model

**Figure 1:**
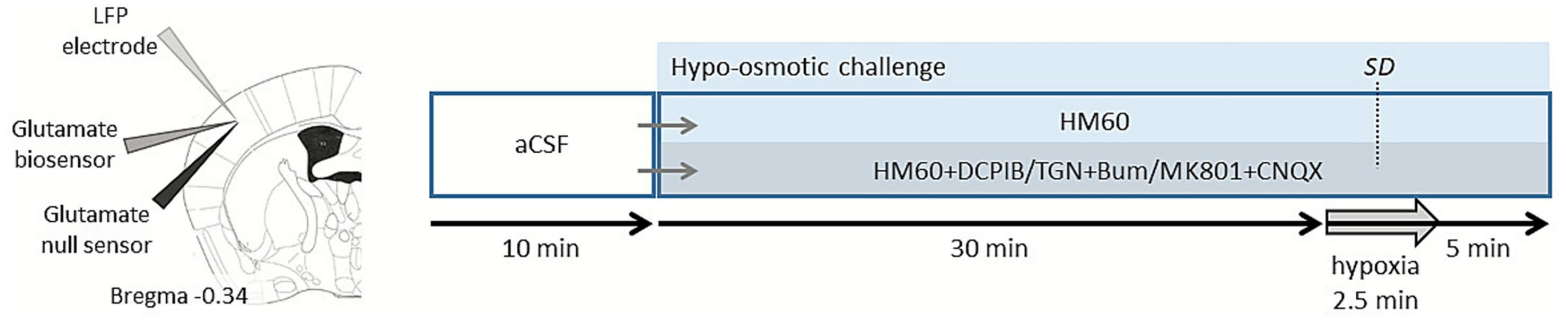
Experimental design and recording setup. A local field potential (LFP) electrode, a glutamate biosensor and a glutamate null sensor were placed in the 3^rd^-4^th^ cortical layers (left). After a 10-min baseline period in artificial cerebrospinal fluid (aCSF), slices were exposed to either hypo-osmotic medium (HM60) alone or HM60 supplemented with DCPIB, or TGN-020 + Bumetanide (TGN + Bum), or MK-801 + CNQX for 30 min. Transient hypoxia (2.5 min) elicited a spreading depolarization (SD), which was followed by a 5 min recovery period (right).

Brain slices were incubated in hypo-osmotic medium (HM), which was prepared by reducing the NaCl concentration of aCSF from the regular 130 to 60 mM (HM60; osmolarity: 199 mosm/l), while other components and the pH of the medium were unaltered (*n* = 17). SDs were elicited by transient hypoxia for 2.5 min (Figure 1).

### 2.3 Local field potential recordings

Local field potential (LFP) measurements filtered in DC mode (<1 Hz) were acquired via a glass capillary microelectrode (1–3 MΩ) filled with 150 mM NaCl and 1 mM HEPES, inserted into the 3^rd^-4^th^ cortical layers (Figure 1). An Ag/AgCl electrode was placed in the recording chamber and served as reference. The microelectrode was connected to a custom-made dual-channel electrometer (including AD549LH, Analog Devices, Norwood, MA, USA) and dedicated differential amplifiers and associated filter modules (NL106 and NL125, NeuroLog System, Digitimer Ltd., United Kingdom). The recorded analogue signal was converted and displayed live using an Acknowledge environment (MP 150, Biopac Systems, Inc.) at a sampling frequency of 1 kHz (Menyhart et al., 2018). The DC potential traces confirmed the occurrence of depolarization events. Further, the DC potential recordings were used off-line to determine SD amplitude, duration at half amplitude (defined as the time between the half-maximal onset and recovery of the negative deflection) and the length of plateau phase (defined as the interval between the maximum negative shift and the beginning of repolarization), from which the plateau duration was calculated.

### 2.4 Measurement of extracellular glutamate concentration

Extracellular glutamate concentrations were acquired together with the DC potential recordings using oxidase enzyme-based microelectrode biosensors (tip diameter: 30–40 μm, length 100 μm) with constant potential amperometry (Vasylieva et al., 2013). Biosensors were constructed of Pt/Ir wires (Goodfellow, Huntington, UK) inserted into a pulled glass capillary (Harvard Apparatus, Edenbridge, UK). The Pt/Ir wire protruding out of the glass capillary was covered with a poly-m-phenylenediamine (PPD) screening layer by electropolymerization (1 h) with 700 mV constant potential and then covered with a bio-layer containing glutamate oxidase and bovine serum albumin (BSA) reticulated with poly(ethylene glycol) diglycidyl ether (Vasylieva et al., 2013). Biosensors were calibrated before and after experiments in 0.01 M phosphate-buffered saline (PBS) pH 7.4 with stepwise injections of glutamate concentration standards (5, 10, 15, 20, 25, 30, 35, 40, 45 μM). Glutamate oxidase enzyme selectivity was confirmed by the application of 5 μM D-serine. During measurements, a constant potential of 500 mV was applied vs. an Ag/AgCl-electrode placed in the recording tissue chamber. Biosensors were implanted adjacent to the LFP microelectrode together with a null BSA sensors (tip diameter: 30–40 μm, BSA, SigmaAldrich, St Quentin Fallavier, France). These control biosensors, coated with BSA, recorded negligible currents (glutamate independent) compared to glutamate biosensors. Biosensors were connected to a dedicated 3-electrode potentiostat (Quadstat) equipped with an eDAQ data acquisition system (eDAQ Pty Ltd., Colorado Springs, CO, USA) and a dedicated eDAQ Chart program. The magnitude of changes in extra-synaptic glutamate concentrations was used to determine glutamate elevation during SD, post-SD glutamate levels, and the area under the curve (AUC; μM × s).

### 2.5 Intrinsic optical signal imaging

For intrinsic optical signal (IOS) imaging, slices were illuminated by a halogen lamp (Volpi AG, Intralux 5,100, Schlieren, Switzerland). Image sequences were captured at 1 Hz with a monochrome CCD camera (spatial resolution: 1024 × 1,024 pixel, Pantera 1 M30, DALSA, Gröbenzell, Germany) attached to a stereomicroscope (MZ12.5, Leica Microsystems, Wetzlar, Germany), yielding 6–10 x magnification. Images were background subtracted and manually thresholded. The total area of depolarization events was then outlined automatically, and corrected manually for area measurements in Fiji. Also, the spatial pattern of SD foci, as well as the propagation velocity of SDs were measured.

### 2.6 Pharmacological treatments

Slices were randomly exposed to various pharmacological treatments during the HM60 application. The aquaporin-4 channel inhibitor 2-(nicotinamide)-1,3,4-thiadiazole (TGN-020; Igarashi et al., 2011; Tocris; 100 μM) was co-applied with the Na-K-Cl cotransporter-1 blocker Bumetanide (Kimelberg and Frangakis, 1985; Sigma-Aldrich; 1 mM; *n* = 13). Another set of slices was exposed to the volume-regulated anion channel (VRAC) blocker 4-(2-Butyl-6,7-dichloro-2-cyclopentyl-indan-1-on-5-yl) oxobutyric acid (DCPIB; Bourke et al., 1981; Tocris; 20 μM; *n* = 15). Finally, NMDA receptors were blocked by the non-competitive NMDA receptor antagonist MK801 (Coan et al., 1987; Tocris; 100 μM), co-applied with the AMPA/kainate receptor antagonist CNQX (Yoshiyama et al., 1995; Tocris; 20 μM; *n* = 8; Figure 1).

### 2.7 Data analysis

LFP and extracellular glutamate concentration were acquired, displayed live, and stored using a personal computer equipped with dedicated software (AcqKnowledge 4.2 for MP 150, Biopac Systems, Inc., USA for LFP; and eDAQ Chart, eDAQ Pty Ltd., Colorado Springs, CO, USA for glutamate concentration). IOS image sequences were analyzed in the image processing package Fiji (ImageJ, National Institute of Health, Bethesda, USA). The rate of SD propagation was determined by placing a 1-mm scale on the cortex and measuring the number of IOS frames required for the SD wavefront to travel this distance, from which propagation velocity was calculated in mm/min. The SD area was assessed manually by delineating the cortical region affected by the SD in the IOS images and normalizing it to the total cortical area. Slice swelling was quantified by measuring changes in the total surface area of the brain slice prior to and with SD relative to baseline area (taken before HM60 administration). Measurements were performed on contrast-enhanced images using Fiji. Data are presented as mean ± standard deviation (stdev). Each data set was first evaluated by a Shapiro–Wilk test of normality to guide the choice of parametric or non-parametric statistics. Data sets with normal distribution were further evaluated with one-way ANOVA followed by a Holm-Sidak *post hoc* test. In case of non-normal distribution, Mann–Whitney Rank Sum Test or Kruskal-Wallis test with Dunn’s post hoc test was carried out. Pearson correlation analysis was performed to assess the relationship between glutamate AUC and SD duration. The level of significance was set at *p* < 0.05*, *p* < 0.01**. Distinct statistical methods are provided in detail in each figure legend.

## 3 Results

### 3.1 Inhibition of cell swelling or ionotropic glutamatergic signaling attenuate the propagation of spreading depolarization under hypo-osmotic stress

**Figure 2:**
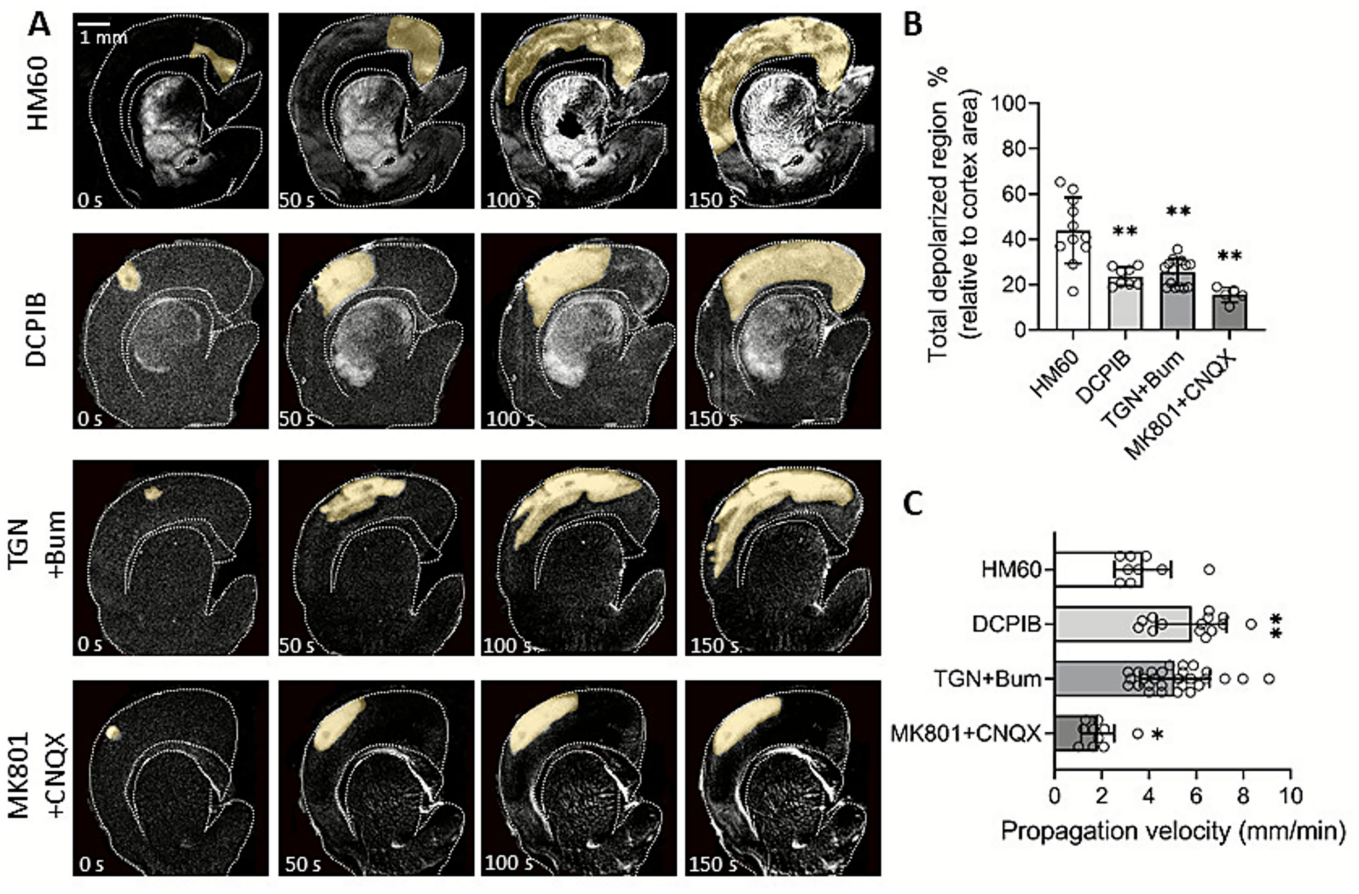
Pharmacological modulation of SD propagation under osmotic stress. **(A)** Representative, background subtracted intrinsic optical signal (IOS) images demonstrate the spatio-temporal evolution of spreading depolarization (SD) in various treatment groups. Yellow shading highlights the propagation of SD in the cortex over time. **(B)** Quantification of the SD-affected cortical region relative to the total cortical area. **(C)** Graphs show the propagation velocity of SD. Data are presented as mean ± stdev, with individual SD events overlaid. After the evaluation of data distribution with Shapiro–Wilk test of normality (Panel B: *p* = 0.560; C: *p* = 0.105), statistical analysis relied on a one-way ANOVA followed by a Holm-Sidak *post hoc* test (B: *p* < 0.01** vs. HM60, C: *p* < 0.05*; *p* < 0.01** vs. HM60).

First, we measured the SD-affected cortical area under osmotic stress. Brain slices exposed to HM60, where SDs invaded nearly the entire cortical area (Figures 2A,B), served as controls for the treated groups. Inhibition of volume regulated glutamate release by DCPIB or co-inhibition of AQP4 channels and the NKCC1s (TGN-020 and Bumetanide) in HM60, caused a significant reduction of the cortical area engaged in SD (45.1 ± 11.64% and 44.82 ± 13.23 vs. 82.36 ± 11.52%; DCPIB and TGN-020 + Bumetanide vs. HM60; Figures 2A,B). Furthermore, the most significant inhibition was achieved through the antagonism of NMDA (MK-801) and AMPA/kainate (CNQX) receptors (28.84 ± 8.38%; MK801 + CNQX in HM60; Figures 2A,B). These findings suggest that (i) swelling enhances the spatial propagation of SD and that (ii) glutamate release, and more specifically, swelling induced glutamate release facilitate the evolution of SD under osmotic stress. Next, we evaluated the propagation velocity of SDs under osmotic stress. Both DCPIB and TGN-020 + Bumetanide treatments promoted the propagation rate of SDs in HM60 (5.8 ± 1.49 and 5.09 ± 1.47 vs. 3.74 ± 1.2 mm/min; DCPIB and TGN-020 + Bumetanide vs. HM60; Figure 2C). Interestingly, the blockade of NMDA and AMPA/kainate receptors known to be predominantly neuronal caused the opposite effect, a significant reduction in SD speed (1.82 ± 0.7 vs. 3.74 ± 1.2 mm/min; MK801 + CNQX in HM60 vs. HM60; Figure 2C; Table 1).

**Table 1:** Treatment-specific effects: DCPIB and TGN-020 + Bumetanide primarily target astrocytes, consistently inhibiting slice swelling, glutamate accumulation, SD area and duration, while paradoxically facilitating SD propagation rate without affecting amplitude.

	DCPIB (VRAC)	TGN-020 + Bum (AQP4 + NKCC1)	MK801 + NCQX (NMDAR+AMPA/kainateR)
Slice swelling	Inhibition	Inhibition	No effect
Glutamate levels	Inhibition	Inhibition	Inhibition
SD invaded area	Inhibition	Inhibition	Inhibition
SD duration	Inhibition	Inhibition	No effect
SD rate of propagation	Facilitation	Facilitation	Inhibition
SD amplitude	No effect	No effect	Increase
Main target	Astrocyte	Astrocyte	Neuron

In contrast, MK-801 + CNQX act primarily on neurons, lowering glutamate levels and inhibiting SD spread and amplitude, but showing no effect on swelling or SD duration.

### 3.2 Osmotic stress enhances the glutamate release coupled to SDs

**Figure 3:**
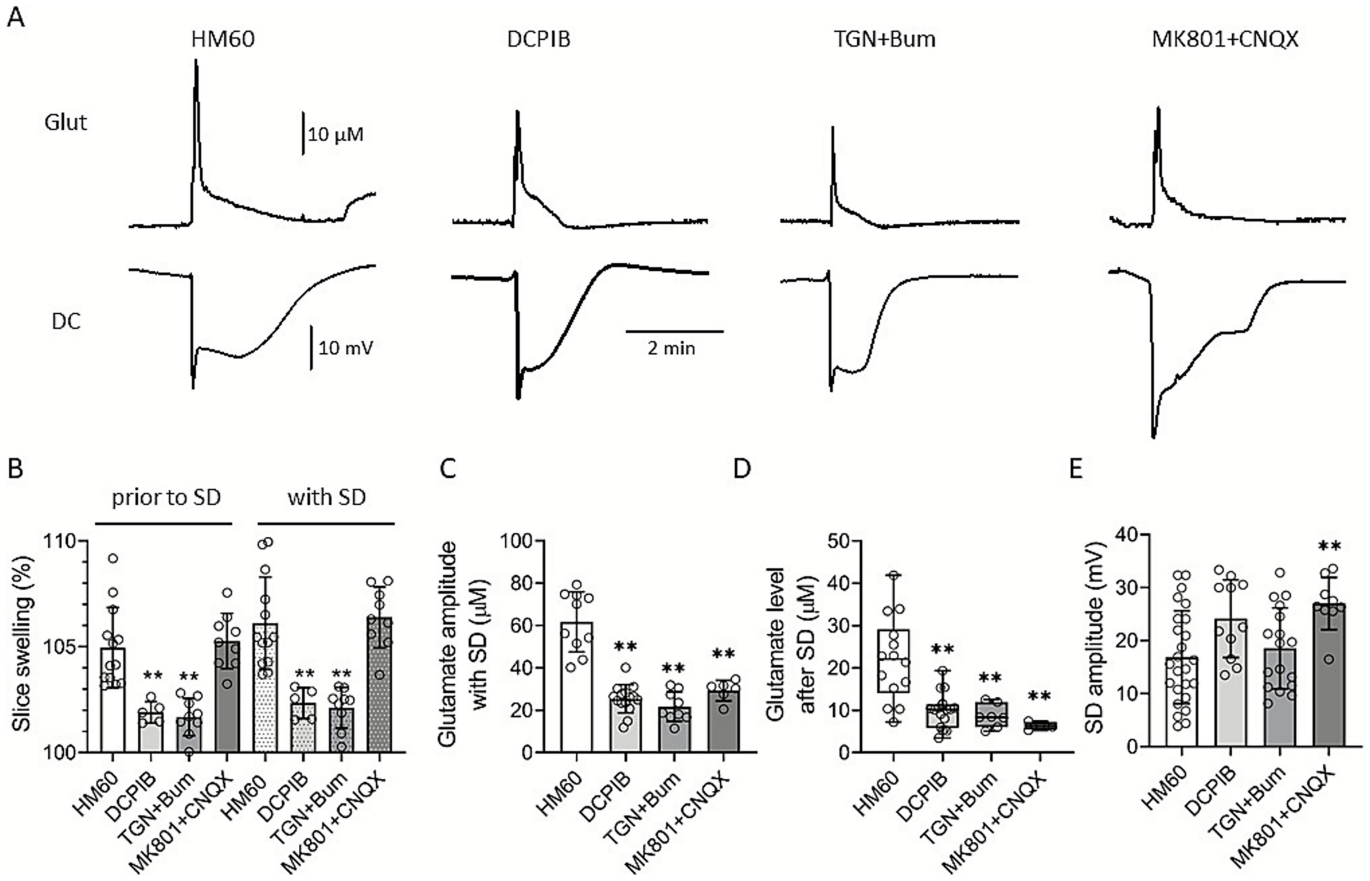
The effect of pharmacological treatments on SD amplitude and extracellular glutamate dynamics. **(A)** Representative electrophysiological recordings of direct current (DC) potential shifts and glutamate transients during spreading depolarization (SD) under different pharmacological treatments. **(B)** Bar charts demonstrate the degree of slice swelling relative to baseline, prior to and with SD, **(C)** extracellular glutamate levels during SD, **(D)** glutamate recovery after SD, and **(E)** SD amplitudes within the treatment groups. Data are presented as mean ± stdev, with individual SD events overlaid. After the evaluation of data distribution with a Shapiro–Wilk test (Panel B: *p* = 0.248; C: *p* = 0.943; D: *p* < 0.050; E: *p* = 0.129), statistical analysis relied on a one-way ANOVA followed by a Holm-Sidak post hoc test in Panels B, C and E (B: *p* < 0.01** vs. HM60; C: *p* < 0.01** vs. HM60; E: *p* < 0.01**vs. HM60). Kruskal-Wallis test with Dunn’s post hoc test was used in Panel D (*p* < 0.01** vs. HM60).

To examine the contribution of astrocytes and neurons to slice swelling, we quantified tissue surface area changes. HM60 induced sizeable slice swelling, which was reduced by DCPIB and TGN-020 + Bum treatments but not by MK801 + CNQX (relative increase in slice surface area: 101.9 ± 0.5, 101.68 ± 0.88, and 105.27 ± 1.3 vs. 104.96 ± 1.89%; DCPIB, TGN-020 + Bumetanide, and MK801 + CNQX vs. HM60). SD further augmented slice swelling, which was also limited by DCPIB and TGN-020 + Bum but not by MK801 + CNQX (102.34 ± 0.73, 102.12 ± 0.96, and 106.39 ± 1.44 vs. 106.11 ± 2.18%; DCPIB, TGN-020 + Bumetanide, and MK801 + CNQX vs. HM60; Figure 3B). To assess SD-associated glutamate accumulation under osmotic stress we measured extracellular glutamate concentrations using enzyme-based microelectrodes as previously reported (Menyhart et al., 2022). A rapid elevation in glutamate levels was detected coinciding with the SD onset (Menyhart et al., 2022). In HM60, glutamate peaked at 61.73 ± 14.16 μM (Figures 3A,C) and remained elevated after SD (21.96 ± 9.98 μM; Figure 3D). All the applied treatments, DCPIB, TGN-020 + Bumetanide and MK801 + CNQX significantly reduced the glutamate peak of SD (25.4 ± 6.62, 21.72 ± 7 and 29.28 ± 4.85 vs. 61.73 ± 14.16 μM; DCPIB in HM60, TGN-020 + Bumetanide and MK801 + CNQX vs. HM60; Figures 3A,C) and attenuated the tissue glutamate accumulation during the recovery phase, 3 min after SD initiation (9.85 ± 4.1, 8.66 ± 2.75 and 6.34 ± 0.9 vs. 21.96 ± 9.98 μM; DCPIB, TGN-020 + Bumetanide and MK801 + CNQX vs. HM60; Figure 3D).

Although all three treatments effectively reduced glutamate levels to a similar degree, SD amplitude responded differently (Figure 3E). Notably, MK801 + CNQX application, despite markedly lowering glutamate accumulation, resulted in a significantly elevated SD amplitude (27 ± 4.9 vs. 16.88 ± 8.76 mV; MK801 + CNQX vs. HM60), suggesting a pivotal role of receptor-mediated excitatory currents in shaping the SD waveform. DCPIB and TGN-020 + Bumetanide, in contrast, did not affect SD amplitude significantly.

### 3.3 Spatiotemporal dynamics of SD correlate with glutamate accumulation

**Figure 4:**
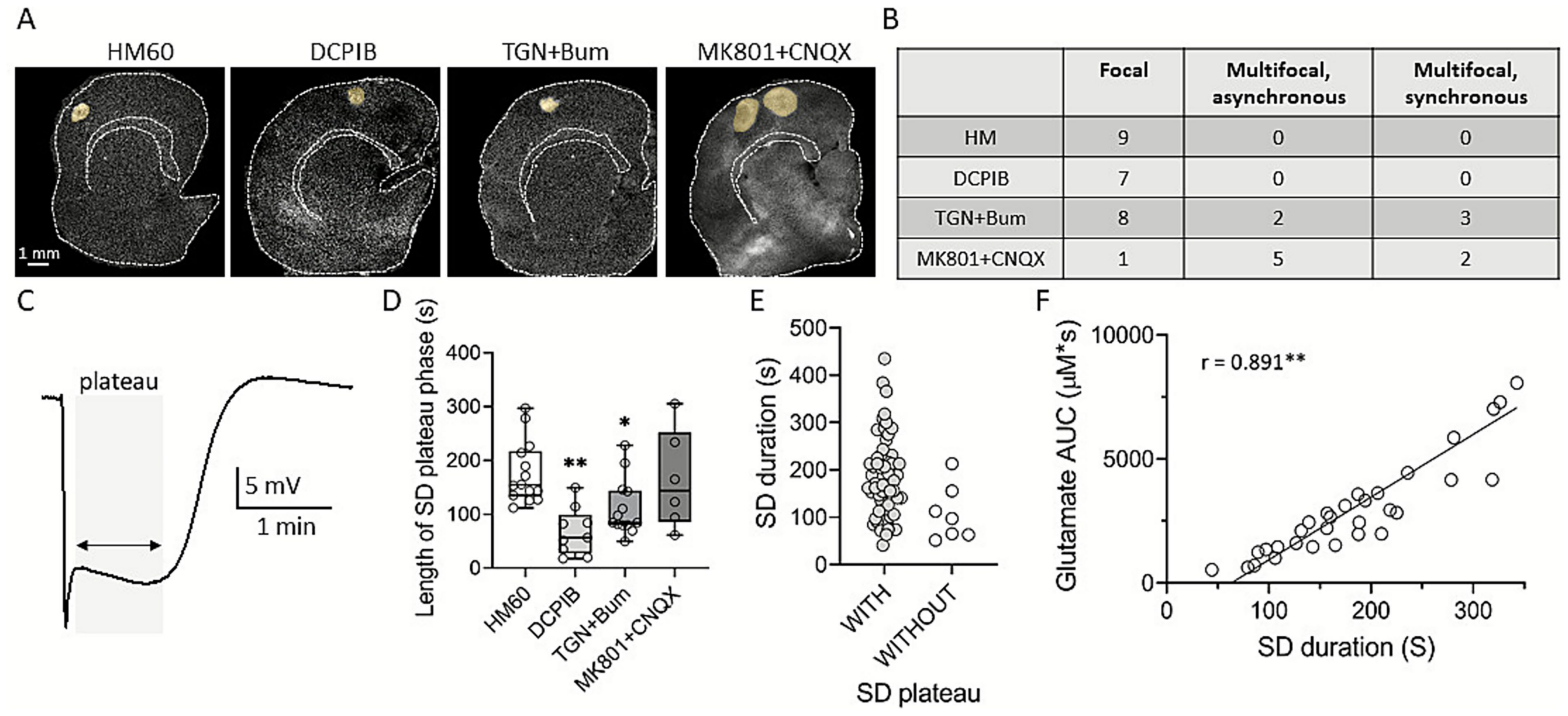
The effect of pharmacological treatments on SD initiation pattern, plateau phase, and glutamate accumulation. **(A)** Representative, background-subtracted intrinsic optical signal (IOS) images illustrate the spatial pattern of SD foci. The SD foci that are typical of each experimental group are highlighted in yellow. **(B)** Incidence of various SD initiation patterns in the experimental groups. **(C)** Representative DC potential recording demonstrating an SD event with a prolonged plateau phase, marked in gray. **(D)** Box plot showing the duration length of SD plateau. **(E)** Scatter plot comparing the duration of SDs with and without a plateau phase, **(F)** correlation of SD duration and glutamate accumulation, expressed as area under the curve (AUC). After the evaluation of normal distribution with a Shapiro–Wilk test (*p* < 0.050), statistical analysis relied on a Kruskal-Wallis test with Dunn’s post hoc test in Panel D (*p* < 0.05* and *p* < 0.01** vs. HM60). In Panel E and F Mann–Whitney Rank Sum Test and Pearson correlation were used.

To further investigate the characteristics of SD under osmotic stress, we analyzed the spatial distribution of SD foci as well as the morphology and duration of the plateau phase of the SD-associated DC potential. In HM60 and DCPIB-treated slices, SDs invariably originated from well-defined focal cortical areas. Under TGN-020 + Bumetanide treatment, 5 of 13 SDs arose from multiple independent foci. In the MK801 + CNQX-treated group, all but one of the eight SDs originated from independent locations within the brain slice (Figures 4A,B). Among multifocal SDs, asynchronous events were defined as those initiated at spatially distinct sites with variable onset times, whereas synchronous events emerged simultaneously from multiple cortical regions (Figures 4A,B).

Our electrophysiological recordings consistently revealed a characteristic feature of the negative DC potential shift of SD wave, known as the “plateau” phase, defined as a sustained negative DC shift following the initial depolarization in contrast to SDs without a plateau, which exhibit only a transient negative deflection (Figure 4C). The plateau phase of SD was invariably present under osmotic stress, and its duration was significantly shortened by both DCPIB and TGN-020 + Bumetanide (67.97 ± 44 and 111.5 ± 52.28 vs. 176.66 ± 57.59 s; DCPIB and TGN-020 + Bumetanide vs. HM60; Figure 4D). SDs lacking a plateau phase—primarily observed in the DCPIB group—also tended to have shorter overall durations (108.37 ± 5,837 vs. 184 ± 83.51 s; SD without plateau vs. SD with plateau; Figure 4E). Finally, we examined the relationship between SD duration and the SD-coupled extracellular glutamate accumulation, and found a strong positive correlation (Pearson r = 0.891; Figure 4F).

## 4 Discussion

Our study provides new insights into the evolution of SDs and the spatial dynamics of their propagation under osmotic stress. We combined (i) intrinsic optical signal (IOS) imaging to characterize the spatiotemporal evolution of SDs, and (ii) electrophysiology with quantitative, enzyme-based amperometry to measure tissue glutamate concentration changes associated with SDs in brain slices exposed to osmotic stress. We show that pharmacological inhibition of cell swelling or ionotropic glutamate receptors significantly attenuates SD-associated glutamate release. Furthermore, we demonstrate that inhibition of tissue swelling shortens SD duration and increases the speed of SD propagation, whereas blockade of ionotropic glutamate receptors slows SD propagation without affecting duration. Together, these findings reveal the complex role of glutamate release in the evolution and propagation of SDs under osmotic stress. For context, we have reported earlier that electrically evoked SDs in normo-osmotic brain slices propagated at lower speed (1.97 mm/min) and arose from a small focal area (0.95 mm^2^) when compared to the SDs observed in hypo-osmolar aCSF (Menyhárt & Frank et al., 2021). In agreement, under hypo-osmotic conditions, SD propagation in our experiments was accelerated and the SD-affected cortical area was larger emphasizing the impact of osmotic stress on SD evolution and its coupling to astrocytic swelling.(Menyhart et al., 2022). In agreement, under hypo-osmotic conditions, SD propagation in our experiments was accelerated and the SD-affected cortical area was larger emphasizing the impact of osmotic stress on SD evolution and its coupling to astrocytic swelling. Importantly, this study was designed to focus on SD initiation and propagation, whereas a detailed analysis of SD recovery—including repolarization, LFP restoration, or K^+^ clearance—was beyond its scope. Future studies incorporating direct K^+^ measurements will be essential to gain a more comprehensive understanding of how astrocyte volume regulation, glutamate signaling, and K^+^ buffering collectively influence SD initiation, propagation, and recovery under osmotic stress.

### 4.1 Inhibition of cell specific glutamate release alters SD-features under osmotic stress

The pharmacological interventions in this study targeted previously described SD-related glutamate release pathways. Based on our prior work and other reports, we applied the VRAC blocker DCPIB and the NKCC1 inhibitor Bumetanide, together with the AQP4 channel antagonist TGN-020, to preferentially target astrocytes. Despite the widespread application of DCPIB for VRAC inhibition it may exert off-target effects at the concentration applied here (20 μM). In addition to blocking VRACs, DCPIB can activate TWIK-related K^+^ channel 1(TREK-1) and inhibit TWIK-related spinal cord K^+^ channel (TRESK; Lv et al., 2019) and has been reported to modulate connexin hemichannels, thus influencing glutamate release and uptake independently of VRAC function (Bowens et al., 2013). These actions may contribute to the observed effect of DCPIB in our study. Therefore, while our findings are consistent with a significant contribution of VRAC-mediated glutamate release to SD evolution under osmotic stress, the effects seen under DCPIB administration cannot be attributed exclusively to VRAC blockade. Astrocytes, which express abundant AQP4 and exhibit robust swelling during brain edema, show markedly reduced volume changes upon AQP4 inhibition. They also maintain higher NKCC1 expression than mature neurons, further supporting astrocyte-specific targeting (Virtanen et al., 2020). In response to swelling, astrocytes release glutamate as an osmolyte to restore physiological volume, a process more prominent in these cells than in other brain cell types (Malarkey and Parpura, 2008; Stokum et al., 2015). Together, the reduction in the peak glutamate extracellular concentration under DCPIB administration likely reflects the effects of primarily glial VRAC inhibition. Notably, however, that when the slice swells, the relative volume fraction of the extracellular space decreases, which mechanically increases extracellular glutamate concentration. Due to this swelling, the measured glutamate peaks here are much higher than those reported earlier (~11 μM), where only neuronal glutamate contributes substantially (Hinzman et al., 2015). VRAC inhibition (DCPIB) and combined AQP4/NKCC1 blockade (TGN-020 + Bumetanide) both significantly reduced the cortical area affected by SD, consistent with earlier reports (Figure 2; Yao et al., 2015; Alvarez-Merz et al., 2022) and our previous findings on astrocyte swelling in SD susceptibility (Menyhart et al., 2022). Inhibition of VRAC or AQP4 and NKCC1 reduced the cortical tissue volume involved in SD extent yet paradoxically accelerated propagation, suggesting distinct astrocyte-dependent mechanisms differentially regulate spatial and temporal SD dynamics (Figure 2). This aligns with prior evidence that astrocytes can either buffer or facilitate synaptic activity depending on local ionic conditions (Bellot-Saez et al., 2017; Ohno et al., 2021; Macaulay, 2021; Zhao et al., 2022). Since neurons primarily drive SD evolution, we tested whether ionotropic glutamate receptors contribute to SD-related glutamate release using a cocktail of the NMDA antagonist MK-801 and the AMPA/kainate antagonist CNQX. Although not the main mediators of glutamate release under osmotic stress, these receptors are proved to be critical for SD propagation. In contrast to our results Basarsky et al., reported previously that both competitive (CGS-17355) and noncompetitive (MK-801) antagonism of NMDA receptors were sufficient to inhibit ouabain-induced SD (Basarsky et al., 1999). Others have also shown that NMDA receptor antagonists reliably prevent SD initiation in normoxic tissue (Nellgard and Wieloch, 1992; Richter et al., 2012). Our results indicate that under hypoosmotic conditions, SD initiation persists despite combined NMDA and AMPA receptor blockade (MK-801 + CNQX), although SD propagation is slowed. Although combined NMDA and AMPA/kainate receptor antagonism slowed SD propagation in our experimental setting, the use of MK-801 + CNQX together prevents us from attributing the observed effects specifically to either NMDA or AMPA/kainate receptors. Therefore, our findings should be interpreted as reflecting the net effect of broad ionotropic glutamate receptor inhibition, without assigning specific roles to individual receptor types. The controversy between our findings and previous NMDA antagonist studies likely reflects fundamental differences in SD elicitation paradigms—metabolic inhibition in their study versus osmotic stress in ours—as well as potential differences in the cellular locus of initiation, which is classically considered neuronal but may shift toward astrocyte involvement under swelling. Although SD initiation is primarily driven by extracellular K^+^ elevation, glutamate also rises during SD (Iijima et al., 1998; Zhou et al., 2013). We observed marked SD-coupled glutamate elevations attenuated by all treatments (Figure 3). This suppression did not reduce SD amplitude; in fact, MK-801 + CNQX increased it, consistent with reports that SD amplitude depends more on extracellular K^+^ than glutamate (Herreras and Somjen, 1993b; Herreras and Somjen, 1993a).

### 4.2 Glutamate dynamics during SDs under osmotic stress

SD-coupled slice swelling was significantly reduced by blocking VRAC or AQP4 and NKCC1, but not by MK-801 + CNQX (Figure 3) highlighting that decreased astrocyte swelling is pivotal in limiting SD-coupled edema, however blocking ionotropic glutamate receptors does not prevent tissue swelling. Our observation fits well with previous work showing that astrocyte swelling, through AQP4 and NKCC1 is tightly coupled to volume regulated astrocytic glutamate release and contributes to SD evolution (Risher et al., 2009; Lafrenaye and Simard, 2019; Hermanova et al., 2024). Glutamate dynamics analysis showed reduced post-SD accumulation with all treatments, suggesting enhanced astrocytic clearance via excitatory amino acid transporter 1/2 (EAAT1/2; Tanaka et al., 1997; Rothstein et al., 1996) or reduced astrocytic glutamate release. In concert, our findings and others also proposed that prolonged glutamate accumulation or impaired clearance heightens excitability and may exacerbate damage upon the formation of edema (Rothstein et al., 1996; Menyhart et al., 2022; Hubel et al., 2017). Moreover, astrocytic IP₃/Ca^2+^ signaling may also modulate neuronal recovery, as studies in IP₃R2 knockout mice demonstrated reduced astrocytic Ca^2+^ signaling and delayed recovery from SD (Monai et al., 2021). These findings indicate that alterations in astrocytic Ca^2+^ pathways can impact the temporal dynamics of SD resolution, potentially through effects on ion and neurotransmitter homeostasis. Integrating these observations with our results, astrocytes appear to influence extracellular glutamate dynamics, Ca^2+^ signaling, and associated K^+^ handling, all of which could affect neuronal excitability under pathological conditions.

While our findings highlight the role of astrocyte glutamate release and volume regulation shaping SD dynamics, we did not directly measure extracellular K^+^ changes in this study. Extracellular potassium serves as a key driver of SD initiation and strongly influences both propagation and recovery. Previous work suggests that astrocytes contribute to K^+^ homeostasis during swelling-associated regulatory volume decrease and Ca^2+^ signaling can affect the timing of SD recovery indicating the interplay between Ca^2+^ signaling, glutamate release and potassium buffering in SD evolution (Basarsky et al., 1999), (Monai et al., 2021).

Curiously, the inhibition of NMDA + AMPA/kainate receptors was most likely to induce multifocal SDs (Figure 4). Such events may also result from disrupted astrocytic gap-junction coupling (Giaume et al., 2010) and impaired network homeostasis, which is thought to be independent of ionotropic receptor signaling. While reduced extracellular glutamate often correlates with less damage, multifocal SDs suggest enhanced synaptic excitability alongside severe astrocytic or ionic dysfunction. Under these conditions, local SDs can emerge independently, indicating that multiple SD foci can occur even at lower extracellular glutamate levels, which may have implications for tissue damage that warrant further investigation. In line with this, tissue swelling might often trigger cytotoxic glutamate elevation and hyperexcitability (Menyhart et al., 2022). Our observation is consistent with the ongoing debate highlighting that SD itself and not the glutamate-mediated excitotoxicty is the major contributor to progressive injury after ischemic stroke (Andrew et al., 2022).

Shortened or absent SD plateaus with DCPIB and TGN + Bum, versus persistence with MK-801 + CNQX, indicate that reduced astrocyte swelling limits SD duration. Longer SDs correlated with greater glutamate AUC, supporting a role for glutamate in sustaining depolarization under osmotic imbalance (Figure 4; Simard and Nedergaard, 2004; Walz, 2000). Moreover, an experimental study carried out in mouse slice cultures after chemical ischemia reported atypical “plume-like” glutamate release events, which contributed to extracellular glutamate accumulation even when network activity was minimal, further supporting the role of dysfunctional astrocytic glutamate clearance in promoting SD propagation under metabolic stress (Ziebarth et al., 2025). Similar glutamate plumes were also observed in a mouse model of familial hemiplegic migraine type 2 (FHM2), where impaired or blocked astrocyte glutamate clearance was associated with higher basal glutamate level and plume frequency, predicting the onset of SD (Parker et al., 2021).

**Figure 5:**
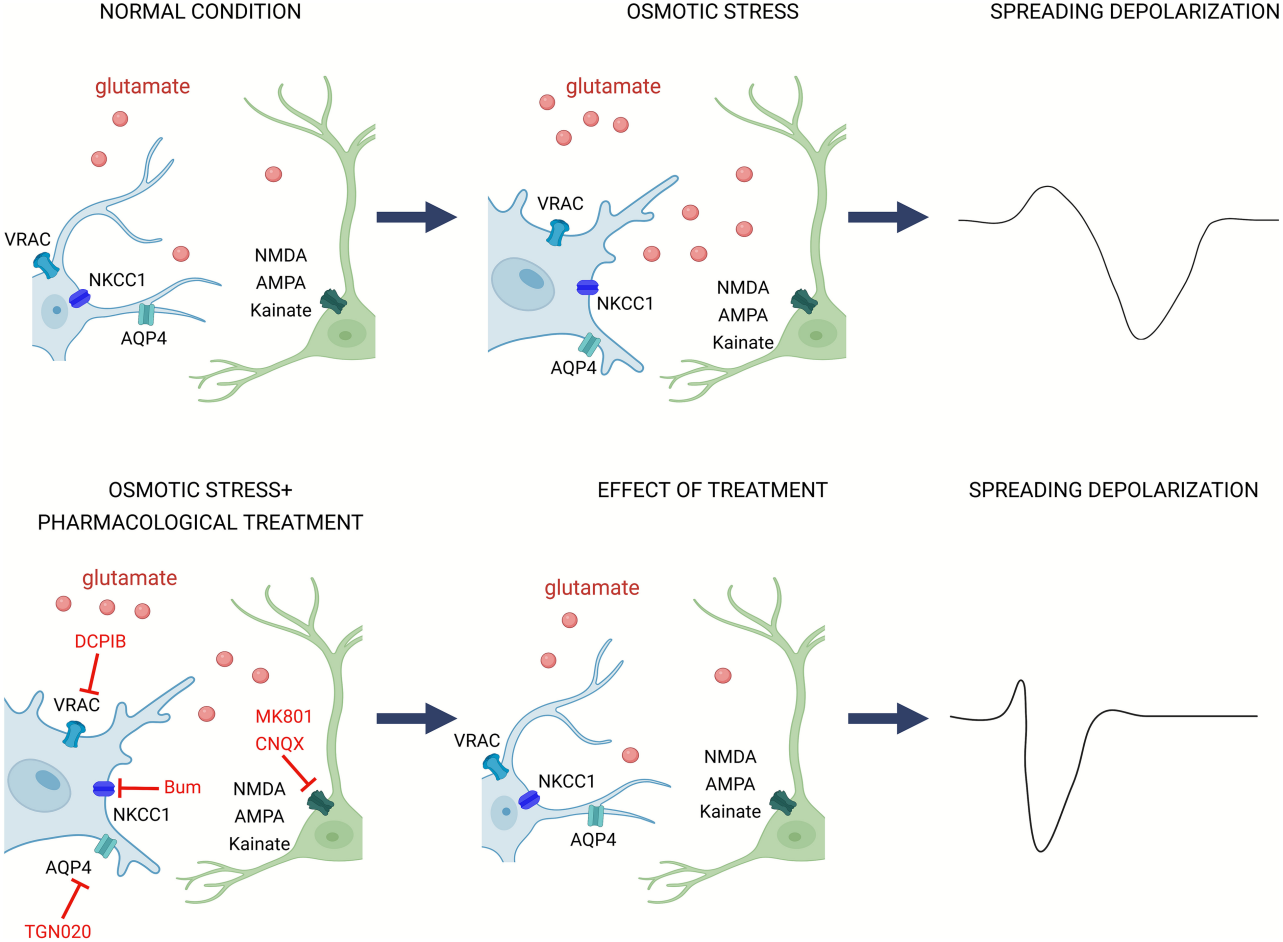
Mechanistic summary of swelling-induced glutamate release and neuronal receptor activation during spreading depolarization. Under physiological conditions, astrocytes maintain extracellular glutamate and osmotic homeostasis via aquaporin-4 channels (AQP4), Na^+^-K^+^-2Cl^−^ cotransporter 1 (NKCC1) and volume-regulated anion channel (VRAC), thereby preserving normal neuronal excitability. Osmotic stress promotes astrocyte swelling which activates VRAC to release glutamate, elevating extracellular glutamate and overstimulating neuronal NMDA N-methyl-D-aspartate (NMDA), α-amino-3-hydroxy-5-methyl-4-isoxazolepropionic acid (AMPA) and kainate receptors—this cascade fosters the occurrence of spreading depolarization. Pharmacological blockade—using DCPIB (VRAC blocker), Bumetanide (NKCC1 inhibitor), TGN-020 (AQP4 inhibitor), along with NMDA antagonist MK-801 and AMPA/kainate antagonist CNQX—attenuates astrocyte swelling, reduces glutamate accumulation and alleviates spreading depolarization. The figure was created in https://BioRender.com.

As summarized in Figure 5, osmotic stress promotes astrocyte swelling and glutamate release and/or accumulation, which in turn interact with neuronal ionotropic glutamate signaling to shape SD evolution. Pharmacological blockade of these pathways reveals their distinct but complementary roles in controlling SD characteristics. Most notably, understanding the interplay between astrocyte water and ion transport mechanisms and neuronal excitability is central to mitigate SD-related injury in stroke.

## 5 Conclusion

Taken together, our data support a model in which astrocyte volume regulation and ionotropic glutamate receptors act synergistically to control SD under hypo-osmotic stress. Astrocytic mechanisms primarily shape the spatial spread and plateau dynamics of SD, whereas glutamatergic transmission governs SD propagation and amplitude. Notably, we show for the first time that these mechanisms can generate multifocal SDs. These findings underscore the critical role of astrocyte–neuron interactions in SD pathology and emphasize that a deeper understanding of the underlying mechanisms is essential for identifying astrocytic water and ion homeostasis as a potential therapeutic target to limit nervous tissue injury.

## Data Availability

The raw data supporting the conclusions of this article will be made available by the authors, without undue reservation.
